# Increased risk of benign paroxysmal positional vertigo in osteoporosis: a nationwide population-based cohort study

**DOI:** 10.1038/s41598-019-39830-x

**Published:** 2019-03-05

**Authors:** Hayoung Byun, Jae Ho Chung, Seung Hwan Lee, Chul Won Park, Eun Mi Kim, Inah Kim

**Affiliations:** 1 Department of Otolaryngology, Hanyang University, Seoul, Korea; 2 Department of Health Sciences, Hanyang University Graduate School, Seoul, Korea; 3 Department of Occupational and Environmental Medicine, Hanyang University, Seoul, Korea

## Abstract

Benign paroxysmal positional vertigo (BPPV) is the most common cause of peripheral vertigo, which results from dislodged vestibular otoliths. Because the otoliths are mainly composed of calcium carbonate, it has been suggested that BPPV may be associated with osteoporosis. We aimed to assess the incidence and recurrence of BPPV in osteoporosis patients using a nationwide population-based cohort study with matched control. We used the database of the National Health Insurance Service of Korea, a universal system covering all 50 million Koreans. Osteoporosis was defined as patients who underwent bone mineral density tests and visited a clinic three or more times between Jan 2004 and Dec 2006. A control cohort consisted of non-osteoporotic subjects socio-demographically matched in a ratio of 1:1. The incidence and recurrence of BPPV between Jan 2007 and Dec 2016 were evaluated. A total of 177,797 osteoporosis patients and the same number of matched controls were identified. The incidence rates (IR) of BPPV in the osteoporosis patients and controls were 31.58 and 18.09 per 1000 persons, respectively (ratio of IR, IRR = 1.75, 95% CI 1.67–1.83). The IRs of recurrent BPPV were 187.3/1000 in the osteoporosis, 163.5/1000 in the controls (IRR = 1.15, 95% CI 1.02–1.28). In multivariate analysis, osteoporosis, female gender (adjusted HR = 1.76), age <65 (adjusted HR = 0.8), living in a metropolis, earning more than the lowest income and hypertension were significantly associated with increased risk of BPPV development. For recurrence, osteoporosis was the only meaningful risk factor (adjusted HR = 1.12). In conclusion, the risks of BPPV development and recurrence are higher in osteoporosis. Physicians might consider informing osteoporosis patients of the risk of developing BPPV and related falls.

## Introduction

Benign paroxysmal positional vertigo (BPPV) is the most common cause of peripheral vertigo, with a lifetime prevalence of over 2.4%^1,2^. The typical characteristics of BPPV are brief recurrent vertigo attacks provoked by head movement^3^. It is believed that BPPV is caused by dislodged fragments of vestibular otoliths that happen to enter the semicircular canals (SCCs). The movement of otoliths in the affected SCC causes an abnormal endolymphatic flow that stimulates or inhibits the vestibular afferent signals and is followed by positional whirling vertigo. As otoliths are composed of inorganic calcium carbonate crystals and protein, calcium metabolism is thought to be involved in the synthesis and resorption of otoliths^4^.

Osteoporosis is a common metabolic disorder characterized by reduced bone mass and deterioration of bony architecture, and resulting from an imbalance between bone formation and resorption. Issues of osteoporosis and related fractures are emerging in our aging society. According to the Korea National Health and Nutrition Examination Survey, the prevalence of osteoporosis in adults aged 50 years or more was 35.5% in women and 7.5% in men in 2008–2009^5^. In the United States, the number of patients with osteoporosis-related hip fractures was 1.3 million people in 1990; it increased by about 25% in the following decade to about 1.6 million in 2000, and is still increasing^6^.

As both osteoporosis and the detachment of otoliths causing BPPV are related to calcium homeostasis, an association between osteoporosis and BPPV has been suggested. Vibert *et al*., in a study of 32 women with BPPV, reported that elderly patients with BPPV had a significantly lower T-score on bone mineral density (BMD)^4^. Yamanaka *et al*., in a case-control study of 61 BPPV patients, found that the incidence of osteoporosis in BPPV patients was similar to that in the general population, whereas the incidence of recurrence was significantly higher in the osteoporosis patients^7^. They also reported that the lower the BMD, the higher the frequency of BPPV recurrence^7^. However, a case-control study of 78 BPPV patients and same number of controls concluded that osteoporosis and vitamin D deficiency were not risk factors for BPPV^3^ and concluded that the coexistence of BPPV with osteoporosis and vitamin D deficiency was coincidental^3^.

As mentioned above, most previous studies examined the incidence of osteoporosis or vitamin D deficiency in BPPV patients. In the present study, on the other hand, the question posed was whether the risk of BPPV was higher in osteoporotic patients. Considering that dizzy patients have an increased risk of falling and osteoporosis-related fractures, this was a question that needed to be answered when considering the relationship between osteoporosis and BPPV.

To answer this question, a population-based retrospective cohort study was designed. The National Health Insurance System (NHIS) of Korea is a universal nationwide coverage system insuring the entire Korean population, whose medical data is officially managed. Using this extensive medical database, a nationwide retrospective cohort study was performed to evaluate the development and recurrence of BPPV in an osteoporosis cohort.

## Materials and Methods

### Study Setting and population

The NHIS of Korea has insured all Koreans since 1989. The information related to medical care is maintained in the NHIS database, which is available for research purposes with formal approval^8^. This study used the National Health Information Database (NHIS-2018-1-230) for medical information since January 2002. The diagnostic classifications followed the 10^th^ revision of the International Statistical Classification of Diseases and Related Health Problems (ICD-10). The authors declare no conflict of interest with the NHIS.

This investigation was approved by the local ethics review board (Hanyang University Guri Hospital Institutional Review Board, GURI 2018-03-012) and performed in accordance with the Declaration of Helsinki and good clinical practice guidelines.

### Study design

**Figure 1:**
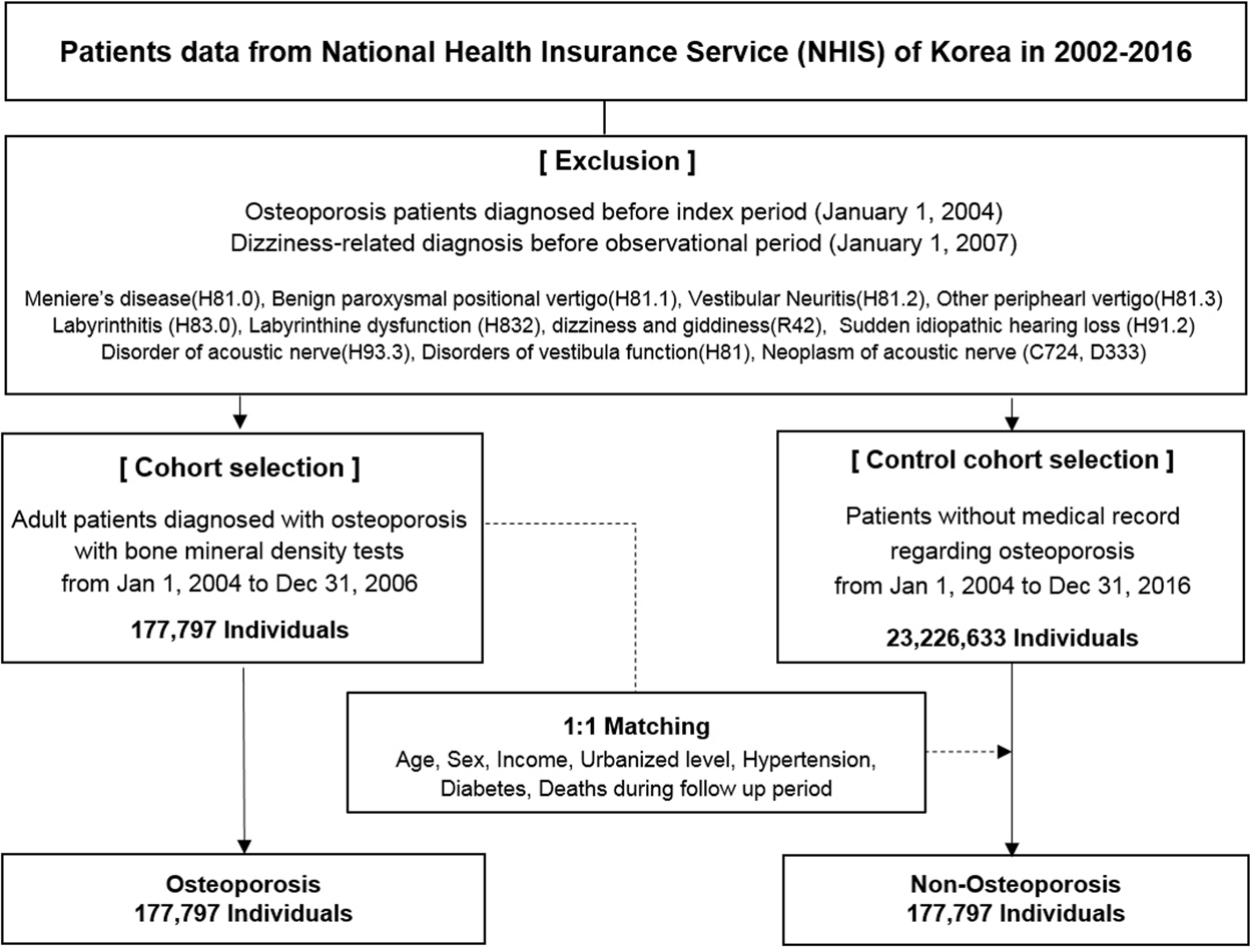
The diagram shows the design of the retrospective cohort study. The osteoporosis cohort included patients who were diagnosed with osteoporosis between January 2004 and December 2006. Each osteoporotic patient was matched with a non-osteoporotic subject. During the follow-up period of 10 years, the development and recurrence of BPPV were assessed in both cohorts.

**Figure 2:**
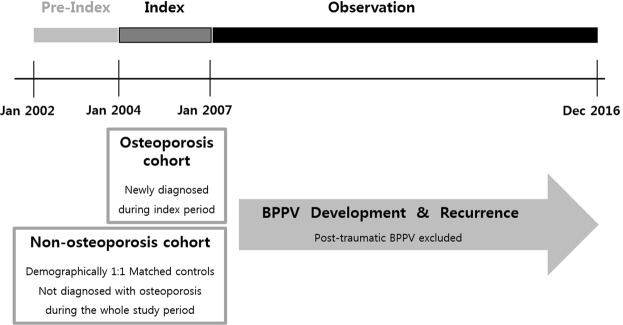
Flowchart of the construction of the cohorts.

A retrospective cohort study was designed (Fig. 1**)**. The cohort group was selected for 3 years of index period from January 2004 to December 2006. During the following observation period from January 2007 to December 2016, the risks of development and recurrence of BPPV were analyzed. As shown in Fig. 2, previously diagnosed osteoporotic patients during the pre-index period, before the index period, and people with any preexisting dizziness-related diagnosis before the observational period were excluded from the initial NHIS population. The patients newly diagnosed with osteoporosis during the index period were selected as the osteoporosis (OSPO) cohort. The control group (N-OSPO) was selected from the population that never received osteoporosis-related medical care during the entire study period from January 2002 to December 2016. Each osteoporotic patient was matched 1:1 with a non-osteoporotic subject for gender, age, hypertension, diabetes, income, and urbanization level.

### Operational definitions of osteoporosis and BPPV

Osteoporosis was defined as patients with the diagnostic code of “osteoporosis” (M80- 82) in ICD -10 who used medical services three times or more and underwent a diagnostic BMD tests such as dual-energy X-ray absorptiometry (DXA), quantitative computed tomography, peripheral DXA, or quantitative ultrasound.

BPPV patients were defined as subjects who received the canalith repositioning treatment with the ICD codes of dizziness [benign paroxysmal positional vertigo (H811), other peripheral vertigo (H813), other disorder of vestibular function (H818, H819), or dizziness and giddiness (R42)]. Recurrence of BPPV was defined as a repeated episode more than 90 days after repositioning treatment. Subjects diagnosed with other inner ear diseases at any point during the study period were excluded from the analysis. These diseases were: Meniere’s disease (H81), Vestibular Neuritis (H812), Labyrinthitis (H83), Labyrinthine dysfunction (H832), Sudden idiopathic hearing loss (H912), Disorder of acoustic nerve (H933), Disorders of vestibular function (H81), and Neoplasm of internal auditory canal (C724, D333). In addition, post-traumatic BPPV was not enrolled, by excluding patients who suffered from BPPV within 30 days of head trauma (S00–S09).

### Statistical analysis

Data were analyzed with the use of SAS Enterprise Guide software version 7.1 (SAS Institute, Inc., Cary, NC). The demographic characteristics of the study population are presented as percentages for categorical variables, and means and standard deviations for continuous variables. Incidence rates (IR) per 1,000 person-years were calculated with 95% confidence intervals. Ratios of IR (IRR) for BPPV occurrence and recurrence were calculated in the OSPO cohort relative to the N-OSPO group. A Cox proportional hazards regression model was used to analyze the risk of BPPV (Hazard ratio, HR), and Kaplan-Meier curves were plotted of the cumulative incidence of BPPV in each cohort. P values < 0.05 were considered to indicate statistical significance.

## Results

### Demographic characteristics of the study population

During the index period, a total of 177,797 patients completed the BMD tests and visited clinics three or more times under a diagnosis of osteoporosis (Fig. 2). The control cohort consisted of subjects matched 1:1 with the OSPO group for age, sex, comorbidity and socioeconomic status. Table 1 shows the demographic characteristics of the cohort groups. The average follow-up periods in the two groups were 8.8 years and 8.7 years, respectively. Women accounted for 91.2% of the total study population and the mean age was 64.5 years.

**Table 1:** Comorbidities and sociodemographic characteristics of the study cohort.

	Osteoporosis (OSPO)		Non-Osteoporosis (N-OSPO)	
	N = 177,797	(%)	N = 177,797	(%)
Gender				
Male	15,731	(8.8)	15,731	(8.8)
Female	162,066	(91.2)	162,066	(91.2)
Age (Mean ± SD)	64.5 ± 11.5		64.5 ± 11.5	
<65	83,195	(46.8)	83,195	(46.8)
≥65	94,602	(53.2)	94,602	(53.2)
Follow-up years (mean ± SD)	8.8 ± 2.5		8.7 ± 2.8	
Income (mean ± SD)	11.3 ± 7.1		11.3 ± 7.0	
Lowest	48,619	(27.3)	48,619	(27.3)
Lower mid	23,060	(13.0)	23,060	(13.0)
Upper mid	36,904	(20.8)	36,904	(20.8)
Highest	69,214	(38.9)	69,214	(38.9)
Urbanization level				
Metropolis	82,863	(46.6)	82,863	(46.6)
Urban	75,475	(42.5)	75,475	(42.5)
Rural	19,459	(10.9)	19,459	(10.9)
Diabetes				
Yes	17,912	(10.1)	17,912	(10.1)
No	159,885	(89.9)	159,885	(89.9)
Hypertension				
Yes	89,122	(50.1)	89,122	(50.1)
No	88,675	(49.9)	88,675	(49.9)
BPPV				
Yes	4,964	(2.8)	2,801	(1.6)
No	172,833	(97.2)	174,996	(98.4)
Recurrence of BPPV				
Yes	876	(17.6)	437	(15.6)
No	4,089	(82.4)	2,364	(84.4)

### Incidence of BPPV development

**Figure 3:**
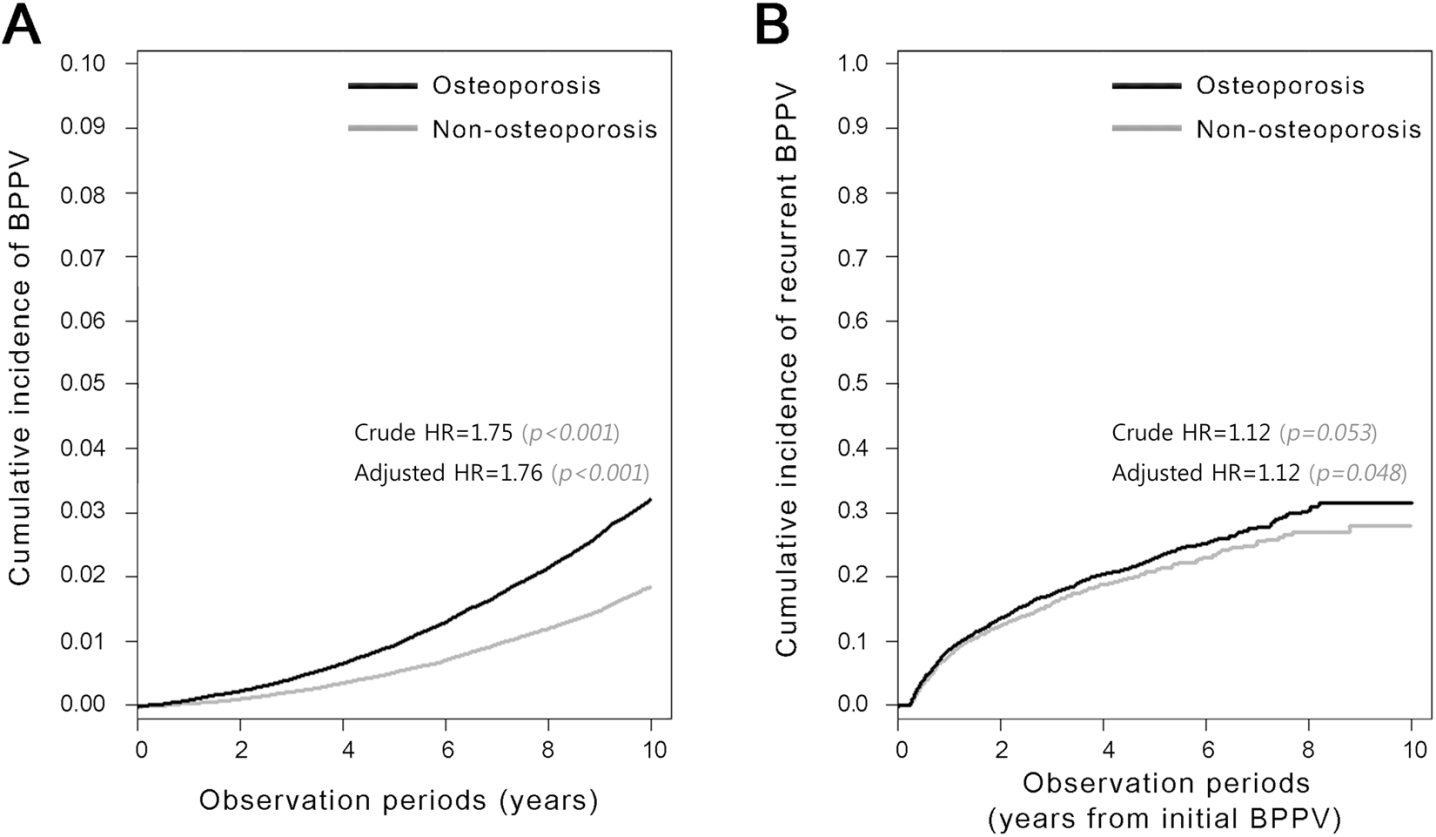
Reverse Kaplan-Meier curves showing the development (**A**) and recurrence (**B**) of BPPV in each cohort. Note that about half of the recurrent cases were diagnosed within 2 years of initial diagnosis (**B**).

In the OSPO cohort, 4964 patients developed BPPV during the observation period (Tables 1, 2). The overall IR of BPPV in OSPO was 31.6 per 1000 persons, 1.75 times higher than that in N-OSPO (18.1 per 1000 persons) (P < 0.001) (Table 2). In both OSPO and N-OSPO, the IR was higher in women than men: 32.7 vs 18.2/1000 in OSPO, 18.6 vs 12.6/1000 subjects in N-OSPO, respectively. In women, BPPV occurred in 2.9% of the OSPO population and 1.6% of the N-OSPO (IRR 1.76, 95% CI 1.68–1.85, P < 0.001). In men, BPPV was observed in 1.3% of the OSPO population and 1.0% of the N-OSPO (IRR 1.44, 95% CI 1.17–1.77, P = 0.005). The reverse Kaplan-Meier curves in Fig. 3A illustrate the occurrence of BPPV in each cohort (Crude HR 1.75, 95% CI 1.67–1.83, P < 0.001).

**Table 2:** The incidence rate (IR) and incidence rate ratio (IRR) of BPPV development in the study cohort.

	Osteoporosis (n = 177,797)					Non-osteoporosis (n = 177,797)					IRR^‡^	95% CI	p
	N	BPPV	Person-Year	IR^†^	95% CI	N	BPPV	Person-Year	IR^†^	95% CI			
Overall	177,797	4,964	1,572,005	31.6	(30.70–32–46)	177,797	2,801	1,548,231	18.1	(17.42–18.76)	**1.75**	(1.67–1.83)	**<0.0001**
Gender													
Male	15,731	219	120,684	18.2	(15.74–20.55)	15,731	151	119,940	12.6	(10.58–14.60)	**1.44**	(1.17–1.77)	**0.0005**
Female	162,066	4,745	1,451,321	32.7	(31.76–33.62)	162,066	2,650	1,428,292	18.6	(17.85–19.26)	**1.76**	(1.68–1.85)	**<0.0001**
**Age**													
<65	83,195	2,838	806,125	35.2	(33.91–36.50)	83,195	1,699	809,096	21.0	(20.00–22.00)	**1.67**	(1.58–1.78)	**<0.0001**
≥65	94,602	2,126	765,880	27.8	(26.58–28.94)	94,602	1,102	739,135	14.9	(14.03–15.79)	**1.86**	(1.73–2.00)	**<0.0001**

^†^IR: incidence rate (per 1000 persons).

^‡^IRR: incidence rate ratio (per 1000 persons).

Statistically significant (P < 0.05) values are highlighted in bold.

### Incidence of BPPV recurrence

The IR of recurrent BPPV in OSPO was 187.3 per 1000 persons, which was 1.15 times higher than that of N-OSPO (163.5 persons per 1000 persons) (P < 0.021) (Table 3). In women, BPPV recurred in 17.7% of OSPO patients and 15.8% in N-OSPO patients (IRR 1.13, 95% CI 1.01–1.28, P = 0.036). In men, the IRR of recurrence did not differ between cases and controls: 16.9% in OSPO, 12.6% in N-OSPO (IRR 1.43, 95% CI 0.82–2.48, P = 0.206). For patients aged 65 years or over, the incidence of recurrent BPPV was higher in the OSPO cohort (IRR 1.23, 95% CI 1.02–1.48). Figure 3B shows the cumulative incidence of recurrent BPPV in each cohort (Crude HR 1.12, 95% CI 0.998–1.26, P = 0.053).

**Table 3:** The incidence rate (IR) and incidence rate ratio (IRR) of BPPV recurrence in the study cohort.

	Osteoporosis (n = 4,964)					Non-osteoporosis (n = 2,801)					IRR^‡^	95% CI	p
	N	Rec BPPV	Person-Year	IR^†^	95% CI	N	Rec BPPV	Person-Year	IR^†^	95% CI			
Overall	4,964	876	46,779	187.3	(174.9–199.7)	2,801	437	26,720	163.5	(148.2–178.9)	**1.15**	(1.02–1.28)	**0.0208**
Gender													
Male	219	37	1,973	187.5	(127.1–248.0)	151	19	1,447	131.3	(72.24–190.3)	1.43	(0.82–2.48)	0.2063
Female	4,745	839	44,806	188.7	(176.0–201.5)	2,650	418	25,273	166.4	(150.5–182.4)	**1.13**	(1.01–1.28)	**0.0356**
Age													
<65	2,838	507	27,001	187.8	(171.4–204.1)	1,699	279	16,322	170.9	(150.9–191.0)	1.10	(0.95–1.27)	0.2075
≥65	2,126	369	19,778	186.6	(167.5–205.6)	1,102	158	10,398	152.0	(128.3–175.6)	**1.23**	(1.02–1.48)	**0.0309**

^†^IR: incidence rate (per 1000 persons).

^‡^IRR: incidence rate ratio (per 1000 persons).

Statistically significant (P < 0.05) values are highlighted in bold.

### Risk factors for BPPV

In a multivariable analysis of the whole cohort, presence of osteoporosis, female gender, age under 65 years, earning more than the lowest income, living in a metropolis and having hypertension were risk factors for development of BPPV (Table 4). The adjusted HR for female gender (1.76, 95% CI 1.68–1.84) was higher than that for osteoporosis (1.54, 95% CI 1.38–1.70). For BPPV recurrence, presence of osteoporosis (adjusted HR = 1.12, 95% CI 1.001–1.26) was a significant risk factor in the whole cohort (Table 4).

**Table 4:** Multivariate analysis of risk factors for the development and recurrence of benign paroxysmal positional vertigo during the follow-up period in the whole cohort.

	Overall development of BPPV (N = 355,594)				Overall recurrence of BPPV (N = 7,765)			
	Crude HR (95% CI)		Adjusted HR (95% CI)		Crude HR (95% CI)		Adjusted HR (95% CI)	
Osteoporosis								
No	1		1		1		1	
Yes	1.75	(1.67–1.83)	**1.76**	**(1.68–1.84)**	1.12	(0.998–1.26)	**1.12**	**(1.001–1.26)**
Gender								
Male	1		1		1		1	
Female	1.60	(1.44–1.77)	**1.54**	**(1.38–1.70)**	1.10	(0.84–1.44)	1.07	(0.82–1.40)
Age								
<65	1		1		1		1	
≥65	0.81	(0.77–0.85)	0.80	(0.76–0.84)	0.93	(0.83–1.04)	0.92	(0.82–1.04)
Income								
Lowest	1		1		1		1	
Lower mid	1.12	(1.04–1.21)	1.08	(1.00–1.16)	0.77	(0.63–0.93)	0.75	(0.62–0.92)
Upper mid	1.18	(1.10–1.26)	1.15	(1.07–1.23)	0.96	(0.82–1.13)	0.95	(0.81–1.12)
Highest	1.20	(1.13–1.27)	1.20	(1.13–1.27)	0.92	(0.80–1.06)	0.93	(0.81–1.07)
Urbanized level								
Metro polis	1		1		1		1	
Urban	0.80	(0.76–0.83)	0.81	(0.77–0.85)	0.95	(0.85–1.06)	0.95	(0.85–1.06)
Rural area	0.57	(0.52–0.63)	0.61	(0.55–0.67)	0.85	(0.67–1.08)	0.84	(0.66–1.07)
Hypertension								
No	1		1		1		1	
Yes	1.05	(1.00–1.10)*	1.13	(1.07–1.19)	0.97	(0.87–1.08)	0.99	(0.88–1.11)
Diabetes mellitus								
No	1		1		1		1	
Yes	1.02	(0.95–1.11)	1.00	(0.92–1.08)	0.95	(0.78–1.16)	0.97	(0.79–1.19)

## Discussion

This study evaluated the significance of osteoporosis as a risk factor for BBPV development and recurrence using nationwide medical data. The findings can be summarized as follows: (1) Osteoporosis patients had a 1.75 times higher risk of developing BPPV than individuals without osteoporosis. (2) The IRR of BPPV recurrence was 1.15, indicating an increased risk of recurrence of BPPV in patients with osteoporosis. (3) The adjusted HRs of osteoporosis for the development and recurrence of BPPV were 1.76 (95% CI 1.68–1.84) and 1.12 (95% CI 1.001–1.26), respectively. To the best of our knowledge, the present study is the first to assess the risk of BPPV and its recurrence in osteoporosis patients using the nationwide population data bank. The results support a positive association between BPPV and osteoporosis.

Otoliths are physiologically contained within the gravity receptor organs of the inner ear. The development, maintenance and degeneration of otoliths are dynamic processes occurring over a lifetime^4^. Dislodged otoliths may travel to the endolymphatic space of the inner ear and enter the SSCs that are responsible for rotational sense. Once in the canals, the otoliths (canaliths) exaggerate endolymphatic flow in response to head rotation, which leads to a spinning sensation. Diagnosis of BPPV can be made on the basis of typical canal-specific positional nystagmus with subjective dizziness^9^. Repositioning of the otoliths from the SCCs to the vestibule is the treatment of choice, and also further supports the diagnosis. Epidemiologic studies show that the prevalence of BPPV is higher in elderly people aged 50–60 years, and the female to male ratio is reported to be 2–3:1^2,3^. Based on the female predominance and possible effect of calcium metabolism on BPPV, there has been much interest in the relationship between BPPV and osteoporosis. A prospective case-control study with 209 subjects found that idiopathic BPPV patients had lower BMDs than controls. In addition, BPPV accompanied by low BMD required more canalith repositioning maneuvers and led to frequent recurrence^10^. Studies of an osteoporotic rat model showed that the density and size of otoliths were different in osteoporotic rats than in the controls^11^ and the authors of that study suggested that these morphological changes might be the consequences of abnormal calcium metabolism related to underlying osteoporosis^11^. However, a systematic review of the relevant literature from 1966 to 2013 concluded that the evidence supporting a possible link between osteoporosis and BPPV was not strong, and the authors noted that further studies were needed to confirm the link^12^.

Another nationwide population-based study analyzing 6649 osteoporotic patients was published during the processing period of the present study. Patients with osteoporosis were found to have a 1.82-fold higher risk of developing BPPV than subjects without osteoporosis, which is similar to our result^13^. However, gender was not found to be a significant risk factor in that study^13^. We used stricter criteria for BPPV, involving not only the diagnostic code but also canalith repositioning therapy and excluding post-traumatic BPPV caused by mechanical damage to the otolith organ. In addition, along with the use of a larger cohort of 177,797 individuals, matching of the control cohort was more accurately performed by considering sociodemographic factors. In addition, we also evaluated the risk of BPPV recurrence in our study. Based on our results, female patients with osteoporosis have a higher risk of developing BPPV (HR 1.54) than males.

It has been reported that the age of peak BPPV incidence is 50–60 years^3^, which is compatible with our data (Tables 2 and 3). Especially in the OSPO cohort, age <65 years was a risk factor for developing BPPV. As to why BPPV is less prevalent over the ages of peak prevalence of osteoporosis, there are several possible explanations. One is that patients over 70 with BPPV frequently experience unsteadiness or imbalance without whirling vertigo and take longer to seek help with their dizziness^14^. This atypical presentation in the elderly may result in less diagnosis of BPPV. Another possible explanation is based on the report that BMD remains nearly constant until the 4th decade and starts to decline rapidly from the 5th decade^15^: the changes related to the rapid decline of BMD may also contribute to otolith detachment followed by BPPV occurrence.

Socioeconomic factors including the household income and urbanization level of individuals were also considered in this study. People who did not earn the lowest level of income and who lived in cities had higher rates of BPPV, which can be explained by the accessibility of medical assistance.

In multivariate analysis, hypertension was a risk factor for BPPV (adjusted HR = 1.13), as shown in Table 4. In view of the previous epidemiologic evidence that low dietary calcium intake significantly increases blood pressure^9,16,17^, this result could be understood as an effect of calcium homeostasis: theoretically a negative calcium balance could affect osteoporosis, hypertension and the otolith environment responsible for BPPV. An association between osteoporosis and hypertension was previously reported in postmenopausal women^18^. Those authors suggested that low dairy calcium intake might increase the risk of both diseases, acting as a possible pathogenic link^18^.

Interestingly, osteoporosis was the only meaningful risk factor for recurrent BPPV (adjusted HR = 1.12) (Table 4). Other factors including gender, age and coexistence of hypertension did not have significant effects. Although its HR for recurrence was lower than for BPPV development, osteoporosis had an independent effect on recurrence (Table 4, Fig. 3).

In South Korea, the medical insurance system was first implemented in 1977 and then expanded to all citizens in 1989. The NHIS of Korea is a single-payer program for the whole nation. In December 2016, 50.763 million (97.1%) of the 52.273 million people covered by the NHIS and another 1.51 million (2.9%), who belong to the lowest income group, were covered by medical aid financed through taxation. In other words, the entire registered Korean population is insured, and the data related to medical care is maintained in the NHIS database. During the 3-year index period of this study, a total of 177,797 osteoporotic patients were newly diagnosed among 42.201 million adult individuals. We used the operational approach of identifying the disease by means of the diagnostic codes (ICD-10) along with the specific examination (BMD) or treatment (canalith repositioning therapy) performed.

This study has several limitations mostly related to the characteristics of the NHIS data, as they do not include laboratory results such as DXA or nystagmography. Because preliminary diagnoses made before performing the laboratory tests have to be excluded, the operational criteria used for diagnosis had to be carefully selected to provide confirmatory diagnoses. Patients had to fulfill three criteria to be diagnosed as osteoporotic: they had to be assigned the appropriate diagnostic code, they had to undergo a diagnostic BMD test, and they had to have used medical services three or more times under the diagnostic code. For the diagnosis of BPPV, patients had to undergo canalith repositioning treatment. Also, to minimize biases, patients that were osteoporotic or dizzy in the pre-index period were strictly excluded (Figs 1 and 2). We note also that no individuals in the control cohort were affected by osteoporosis during the entire study period. Other limitations are that the severity of osteoporosis as indicated by T or Z scores, as well as treatment compliance, could not be assessed in this study. We anticipate that future studies employing prospective cohorts will provide further detailed insight into the relation between osteoporosis and BPPV.

Osteoporosis-related fractures may lead to a reduced quality of life, disability, and even death. Moreover, the direct and indirect medical costs of osteoporosis and its related fractures are massive^19^. Management of osteoporosis is an important issue in primary health care in order to minimize the development of fractures and decrease the social burden. As the present study shows, patients with osteoporosis also have higher risk of BPPV development and recurrence. Healthcare providers should consider implementing effective educational interventions about BPPV for osteoporotic patients since the latter, especially those that are elderly, may have a greater tendency to suffer falls^20^.

## Conclusions

Osteoporosis patients have higher risks of BPPV occurrence and recurrence. Medical counseling concerning BPPV should be considered for individuals with osteoporosis.

## References

[1] Bhattacharyya N (2008). Clinical practice guideline: benign paroxysmal positional vertigo. Otolaryngology–head and neck surgery: official journal of American Academy of Otolaryngology-Head and Neck Surgery.

[2] von Brevern M (2007). Epidemiology of benign paroxysmal positional vertigo: a population based study. Journal of neurology, neurosurgery, and psychiatry.

[3] Kim JS, Zee DS (2014). Clinical practice. Benign paroxysmal positional vertigo. The New England journal of medicine.

[4] Vibert D, Kompis M, Hausler R (2003). Benign paroxysmal positional vertigo in older women may be related to osteoporosis and osteopenia. The Annals of otology, rhinology, and laryngology.

[5] Choi YJ, Oh HJ, Kim DJ, Lee Y, Chung YS (2012). The prevalence of osteoporosis in Korean adults aged 50 years or older and the higher diagnosis rates in women who were beneficiaries of a national screening program: the Korea National Health and Nutrition Examination Survey 2008–2009. Journal of bone and mineral research: the official journal of the American Society for Bone and Mineral Research.

[6] Johnell O, Kanis JA (2006). An estimate of the worldwide prevalence and disability associated with osteoporotic fractures. Osteoporosis international: a journal established as result of cooperation between the European Foundation for Osteoporosis and the National Osteoporosis Foundation of the USA.

[7] Yamanaka T (2013). Osteoporosis as a risk factor for the recurrence of benign paroxysmal positional vertigo. The Laryngoscope.

[8] Song SO (2014). Background and data configuration process of a nationwide population-based study using the korean national health insurance system. Diabetes & metabolism journal.

[9] von Brevern M (2015). Benign paroxysmal positional vertigo: Diagnostic criteria. Journal of vestibular research: equilibrium & orientation.

[10] Jang YS, Kang MK (2009). Relationship between bone mineral density and clinical features in women with idiopathic benign paroxysmal positional vertigo. Otology & neurotology: official publication of the American Otological Society, American Neurotology Society [and] European Academy of Otology and Neurotology.

[11] Vibert D (2008). Ultrastructural changes in otoconia of osteoporotic rats. Audiology & neuro-otology.

[12] Yu S, Liu F, Cheng Z, Wang Q (2014). Association between osteoporosis and benign paroxysmal positional vertigo: a systematic review. BMC neurology.

[13] Chan KC, Tsai YT, Yang YH, Chen PC, Chang PH (2017). Osteoporosis is associated with increased risk for benign paroxysmal positional vertigo: a nationwide population-based study. Archives of osteoporosis.

[14] Batuecas-Caletrio A (2013). Benign paroxysmal positional vertigo in the elderly. Gerontology.

[15] Park EJ (2014). Prevalence of osteoporosis in the Korean population based on Korea National Health and Nutrition Examination Survey (KNHANES), 2008-2011. Yonsei medical journal.

[16] Lundberg YW, Xu Y, Thiessen KD, Kramer KL (2015). Mechanisms of otoconia and otolith development. Developmental dynamics: an official publication of the American Association of Anatomists.

[17] Karatas A (2017). Association of Benign Paroxysmal Positional Vertigo with Osteoporosis and Vitamin D Deficiency: A Case Controlled Study. The journal of international advanced otology.

[18] Varenna M (2013). The association between osteoporosis and hypertension: the role of a low dairy intake. Calcified tissue international.

[19] Dempster DW (2011). Osteoporosis and the burden of osteoporosis-related fractures. The American journal of managed care.

[20] Jumani K, Powell J (2017). Benign Paroxysmal Positional Vertigo: Management and Its Impact on Falls. The Annals of otology, rhinology, and laryngology.

